# High Energy Intake Induced Overexpression of Transcription Factors and Its Regulatory Genes Involved in Acceleration of Hepatic Lipogenesis: A Rat Model for Type 2 Diabetes

**DOI:** 10.3390/biomedicines7040076

**Published:** 2019-09-27

**Authors:** Suresh P. Khadke, Aniket A. Kuvalekar, Abhay M. Harsulkar, Nitin Mantri

**Affiliations:** 1 Interactive Research School for Health Affairs, Bharati Vidyapeeth Deemed University, Katraj, Pune, Maharashtra 411043, India; spkhadke@gmail.com (S.P.K.); kuaniket@gmail.com (A.A.K.); 2 Department of Pharmaceutical Biotechnology, Poona College of Pharmacy, Bharati Vidyapeeth Deemed University, Erandwane, Pune, Maharashtra 411038, India; abhay.harsulkar@gmail.com; 3 The Pangenomics Group, School of Science, RMIT University, Melbourne, VIC 3000, Australia

**Keywords:** diabetes, dyslipidemia, high fat diet, insulin resistance, T2DM

## Abstract

Type 2 diabetes mellitus (T2DM) is a metabolic disorder characterized by impaired insulin action and its secretion. The objectives of the present study were to establish an economical and efficient animal model, mimicking pathophysiology of human T2DM to understand probable molecular mechanisms in context with lipid metabolism. In the present study, male Wistar rats were randomly divided into three groups. Animals were fed with high fat diet (HFD) except healthy control (HC) for 12 weeks. After eight weeks, intra peritoneal glucose tolerance test was performed. After confirmation of glucose intolerance, diabetic control (DC) group was injected with streptozotocin (STZ) (35 mg/kg b.w., i.p.). HFD fed rats showed increase (*p* ≤ 0.001) in glucose tolerance and HOMA-IR as compared to HC. Diabetes rats showed abnormal (*p* ≤ 0.001) lipid profile as compared to HC. The hepatocyte expression of transcription factors *SREBP-1c* and *NFκβ,* and their target genes were found to be upregulated, while *PPAR-γ, CPT1A* and *FABP* expressions were downregulated as compared to the HC. A number of animal models have been raised for studying T2DM, but the study has been restricted to only the biochemical level. The model is validated at biochemical, molecular and histopathological levels, which can be used for screening new therapeutics for the effective management of T2DM.

## 1. Introduction

Non-communicable diseases (NCDs), also known as chronic diseases, result from a combination of genetic, physiological, environmental and lifestyle factors. Among all NCDs, Type 2 diabetes (T2DM) is the most common which accounts for 90% of all forms of diabetes with a rapidly rising prevalence globally. In 2014, the number of individuals with diagnosed diabetes reached close to 387 million with projected figures reported to be 600 million by 2035 [1,2,3]. Around half of diabetics are undiagnosed which results in uncontrolled diabetes and further increase in the global burden of diabetes [2,3,4]. NCDs are one of the major public health challenges of the 21^st^ century, associated with elevated economic burden especially in low- and middle-income countries. Social and economic costs of NCDs continue to escalate at an overwhelming rate and will soon exceed the capabilities of countries to address and manage the burden of these conditions [5].

Type 2 diabetes mellitus is a metabolic disorder characterized by impaired insulin action (insulin resistance), followed by compensatory mechanism of beta cells that secrete excess basal insulin (hyperinsulinemia) to maintain glucose homeostasis. This in turn, leads to in pancreatic beta cell dysfunction. Insulin resistance is a metabolic defect primarily associated with resistance to insulin-mediated glucose disposal. Over time, beta cell function impairment eventuates leading to an imbalance in glucose homeostasis with subsequent development of impaired glucose tolerance and frank diabetes [6,7,8]. Previous reports have indicated that insulin resistance contributes to dyslipidemia associated with type 2 diabetes [9,10]. Elevated serum glucose, triglycerides and cholesterol as well as hypertension and evidence of insulin resistance contribute to the pathogenesis of T2DM and associated complications [11].

Despite recent advances in the prevention and management of diabetes, the alarmingly high morbidity and mortality of this disease ranks second to cardiovascular disease [12]. The development and screening of new preventive and treatment strategies for chronic conditions are largely reliant on initial preclinical evaluation using animal models that mimic the pathogenesis of human disease. Despite complications of the diseases, animal models in preclinical setting are very effective at investigating associated complications and comorbidities, as well as therapeutic interventions. Although several animal models exist for T2DM, their application relies on the components of the disease being studied. For development of hyperglycemia, streptozotocin (STZ) and alloxan models are most commonly used on animals [13,14,15]. In these models, pancreatic beta cells are selectively destroyed, resulting in insulin deficiency rather than the outcome of insulin resistance with resultant characteristics of human type 1 rather than type 2 diabetes [16,17]. Diabetes and its associated complications have to be addressed simultaneously for finding more effective treatments and preventive strategies. It is as important to characterize clinically relevant experimental models for understanding the molecular basis, pathogenesis and mechanism of actions of these therapeutic agents [15].

Smaller size, ease of handling, omnivorous nature, non-wild tranquil behavior, lower cost, wider availability, easy induction of diabetes, and easier maintenance make rodents a model organism compared to the genetic models with deliberately induced defects in one or more genes [18]. High-fat diets (HFD) are commonly used to develop models for metabolic syndrome and their associated complications [3,19,20]. However, these models require expensive diet and lengthy feeding regimens before any detectable decline of mass of β-cells. In one model, this barrier is overcome using streptozotocin (STZ) which depletes β-cell mass experimentally after development of diet-induced insulin resistance [19,21]. Srinivasan et al. [8] reported that rats fed with high-fat diet combined with streptozotocin developed a much closer condition to type 2 diabetes than previous models which mimicked human pathology of disease. Similar studies were conducted by several authors [22,23] but they were all restricted to biochemical analysis of the model.

For development of an efficient model, several investigators have used different sources of carbohydrate (fructose, sucrose), fats, proteins and cholesterol in rodents. Combining different components in preparation of HFD leads to increased cost. Hence, the search for development of ideal animal model for T2DM either by way of modification of the existing methods or by developing new methodologies or a combination of both, continues [8,17,19]. Therefore, the aim of this study is to develop a rodent model that mimics human T2DM closely and validate this model at molecular level. In the present study, animal derived-fat (lard oil) is used in the preparation of the high fat diet (HFD). The animal model is developed using HFD with low dose of streptozotocin and it is validated at the biochemical and molecular level.

## 2. Materials and Methods

### 2.1. Animals

This study was approved by Institutional Animal Ethics Committee (IAEC) of Bharati Vidyapeeth Deemed University, Pune, India (BVDUMC/443/2012–2013, approved on (7 March 2012). For this study, male albino Wistar rats (120–150 gm, 10–11 weeks) were housed in standard animal house conditions (temperature 22 ± 2 °C, 12:12 h light and dark cycle, and 55 ± 5% humidity). During the study period, animals were kept in plastic cages with ad libitum access to feed and water. After acclimatization, animals were randomly assigned to three groups (*n* = 6) as follows:

Group I received normal diet for 12 weeks and was designated as healthy control (HC). Group II and III received high fat diet for the same period. The high fat diet composition is depicted in Table S1. Group II was designated as HFD control (HFDC) while group III was treated with a single low-dose STZ (DC).

The High Fat Diet (HFD) was prepared in our laboratory by using normal pellet diet and 30% lard oil. Normal rat pellet diet was powdered by grinding, mixed with the lard oil (30%) and purified water as per requirement for the preparation of pellets. The mixture was then made into a pellet form.

### 2.2. Experimental Design

Animals from the three groups were kept on designated diets for 12 weeks. After eight weeks, intra peritoneal glucose tolerance test (IPGTT) was performed to detect development of glucose intolerance in rats. After confirmation of glucose intolerance, animals from DC group were injected with STZ (35 mg/kg b.w., i.p.). Food and water intake was recorded daily. Intermediate blood was collected from retro orbital plexus of animals for different biochemical investigation. After completion of the experiment (four weeks), all animals were sacrificed. The blood was collected by cardiac puncture. Liver, pancreas and kidney tissues were excised, snap frozen immediately in liquid nitrogen and stored at −80 °C for further studies. A small portion of the tissues were stored in 10% neutral buffered formalin for histological study.

### 2.3. Intraperitoneal Glucose Tolerance Test (IPGTT)

After 6 hr fasting, blood glucose levels were estimated and glucose solution (2 gm/kg b.w., i.p.) was administered to animals. Blood glucose was estimated using Accu-Chek monitor (Roche Diagnostics Pty. Ltd., Basel, Switzerland, ) at different time points (i.e., 0, 15, 30, 60, 90 and 120 min) from tail vein.

### 2.4. Calculation of Homeostasis Model Assessment of Insulin Resistance (HOMA-IR)

Insulin was measured with rat-specific ELISA kits (Ray Biotech, GA, USA). The HOMA-IR (homeostasis model assessment of insulin resistance) was calculated as per formula from Bhandari et al. [24]:

HOMA-IR = Insulin (μU/mL) × glucose (mM)/22.5(1)

### 2.5. Estimation of Blood Biochemical Measurements

Estimation of glucose, lipid profile, kidney function and liver function were performed from serum. Lipid parameters measured included total cholesterol (TC), triglycerides (TGs), low-density lipoprotein (LDL), very low density lipoprotein (VLDL) and high density lipoprotein (HDL). Creatinine and urea assays were performed to assess the kidney function. Serum glutamic oxaloacetic transaminase (SGOT) and serum glutamic pyruvic transaminase (SGPT) and total protein were also measured. Commercially available kits (Coral Clinical Systems, Goa, India) were used to estimate the biochemical parameters.

### 2.6. Fatty Acid Metabolic Genes and Regulatory Element Selection for Quantitative RT-PCR

Transcription factors viz. Sterol Regulatory Element Binding Protein-1c (*SREBP-1c*), Peroxisome Proliferator-Activated Receptor Gamma (*PPAR-γ*) and Nuclear Factor-κβ (*NFκβ*), which regulate fatty acid metabolism genes, such as Fatty Acid Synthase (*FASN*), Acetyl-CoA Carboxylase Alpha (*ACACA*), Malonyl CoA: ACP Acyltransferase (*MCAT*), Carnitine Palmitoyltransferase 1A (*CPT1A*) and Acyl-CoA Synthetase Long-chain family member 1 (*ACSL1*) and inflammatory marker, Tumor Necrosis Factor - Alpha (*TNF-α*) were selected for further study. A candidate gene, Fatty Acid Binding Proteins (*FABP*) from a family of transport proteins for fatty acids was also selected for the study. KicqStart^®^ Primers were purchased from Sigma Aldrich (New York, USA). The primer sequences are listed in Table S2.

### 2.7. Hepatic Gene Expression Study by Quantitative Real-Time Polymerase Chain Reaction (qRT-PCR)

Hepatic RNA was extracted by using TRIZOL method (Invitrogen, Carlsbad, CA, USA) for the gene expression studies. The isolated RNA quality was assessed by agarose gel electrophoresis (BioRad, Hercules, California, USA). The quantification of RNA was done by ND-1000 UV spectrophotometer (Nanodrop Technologies, Wilmington, DE, USA). For qRT-PCR analysis, cDNA was synthesized from 2 µg of total RNA using the M-MLV reverse transcriptase kit (Invitrogen).

The qRT-PCR was performed with SYBr green expression assays (Applied Biosystems, Waltham, Massachusetts, CA, USA) on StepOne real-time PCR system (Applied Biosystems, Waltham, Massachusetts, CA, USA). In qRT-PCR study, initial denaturation was performed at 95 °C for 10 min. This step was followed by the 40 cycles of 95 °C for 3 s (denaturation,); 60 °C for 30 s (annealing) and at 95 °C for 15 s (extension). The final extension step was performed at 60 °C for 0.15 s. The relative amounts of RNA were normalized to the amount of the endogenous housekeeping control, *GAPDH* (Glyceraldehyde-3-Phosphate Dehydrogenase).

### 2.8. Histopathological Examinations of Various Tissues and Estimation of Tissue Injury by Densitometry

After completion of the experiment (12 weeks), the animals were sacrificed and liver, kidney and pancreas tissues were collected for further analysis. These tissues were instantly fixated in 10% neutral buffered formalin solution (pH 7). Paraffin blocks were then prepared and cross sections (4 µm thick) stained with hematoxylin and eosin. Tissue sections were viewed under light microscope (EVOS™ FL Auto 2 Imaging System, Invitrogen, USA).

Staining intensity and tissue injury was estimated as per the Campbell’s protocol [25] with some modifications. From each tissue, six images were calibrated for tissue injury using Image J software (National Institutes of Health, Bethesda, Rockville, city, MD, USA). The average value of densitometric analysis of tissue injury was evaluated for liver tissues, kidney tissues and beta cells death by measuring stain intensity.

### 2.9. Statistical Analysis

Data are presented as Mean ± Standard Deviation (SD). Statistical analysis was performed using one-way analysis of variance (ANOVA) followed by Dunnett’s Multiple Comparison Test using GraphPad Instat (Version 5, GraphPad Software Inc., San Diego, CA, USA).

## 3. Results

In the present study, high fat diet feed with low streptozotocin induced model was developed to study diabetic dyslipidemia. The model was evaluated at both biochemical and molecular levels.

### 3.1. Standardization of High Fat Diet (HFD)

Effective concentration of the lard oil was determined by addition of 10, 20 and 30% of lard oil to normal powdered diet and results are depicted in Figure S1. The diet containing 10 and 20% lard oil did not produce sustained increase in glucose intolerance and blood glucose. The glucose intolerance was successfully developed at 30% lard oil diet. After confirmation of glucose intolerance, 30% lard oil diet was used in the present study.

The combination of 30% lard oil containing high fat diet together with single low-dose STZ (35 mg/kg b.w.) produced consistent elevated levels of blood glucose in animals throughout the period of experiments.

### 3.2. Animal Body Weight

The body weight of animals during experiment is shown in Figure S2. Diabetic control group rats showed significant difference in body weight as compared to HC (*p* < 0.01) and HFDC (*p* < 0.001).

### 3.3. All Groups Showed Non-Significant Differences in Serum Profile Initially

Before initiation of experiment, baseline values of blood parameters were depicted in Table S3. Initially, all groups showed non-significant difference in serum glucose, lipid profile, kidney and liver function tests.

### 3.4. Assessment of Insulin Resistance After Eight Weeks (Before STZ Induction)

#### 3.4.1. HFD Fed Animals Exhibited Glucose Intolerance

**Figure 1:**
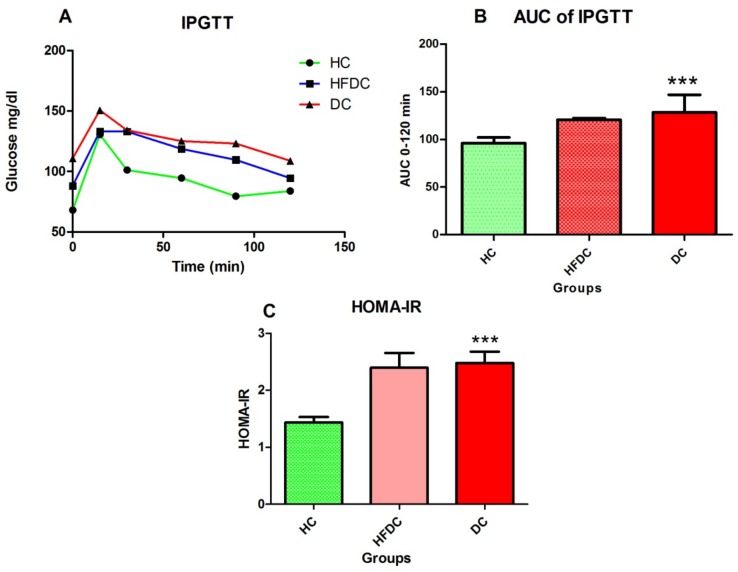
Intra peritoneal glucose tolerance test (IPGTT), area under curve (AUC) and HOMA-IR model for the experimental groups. (**A**) Variations in glucose levels during IPGTT. (**B**) Area under curve (AUC) for IPGTT. (**C**) Homeostasis model assessment of insulin resistance (HOMA-IR). Results are recorded as the mean of three replicates and denoted as mean ± SD (*n* = 6 animals per groups). *** *p* ≤ 0.001, when compared with the healthy control group (Dunnett’s Multiple Comparisons Test). HC: Healthy control, HFDC: High fat diet control, DC: Diabetes control.

Figure 1A,B demonstrates variations in glucose levels and area under curve (AUC) after IPGTT. After eight weeks, IPGTT and HOMA-IR were assessed from all experimental groups. Healthy control rats showed faster serum glucose clearance than HFDC and DC rats. HFDC and DC rats exhibited higher serum glucose levels at 0 and 120 min as compared to the healthy control rats. HFDC and DC also showed significant increases in AUC compared to HC (*p* ≤ 0.01 for HFDC; *p* ≤ 0.001 for DC) groups.

#### 3.4.2. Homeostasis Model Assessment of Insulin Resistance (HOMA-IR)

Serum glucose and insulin levels were depicted in Table 1. After eight weeks HFD fed groups showed significant (*p* ≤ 0.001) increase in serum glucose and insulin (*p* ≤ 0.01, *p* ≤ 0.001) levels as compared to HC control. HOMA-IR of experimental groups is depicted in Figure 1C. HFDC and DC groups showed significant (*p* ≤ 0.001) increase in HOMA-IR as compared to HC group.

### 3.5. Estimation of Serum Biochemical Parameters after Twelve Weeks (After STZ induction)

#### 3.5.1. Detection of Elevated Serum Glucose Levels in DC Group

**Figure 2:**
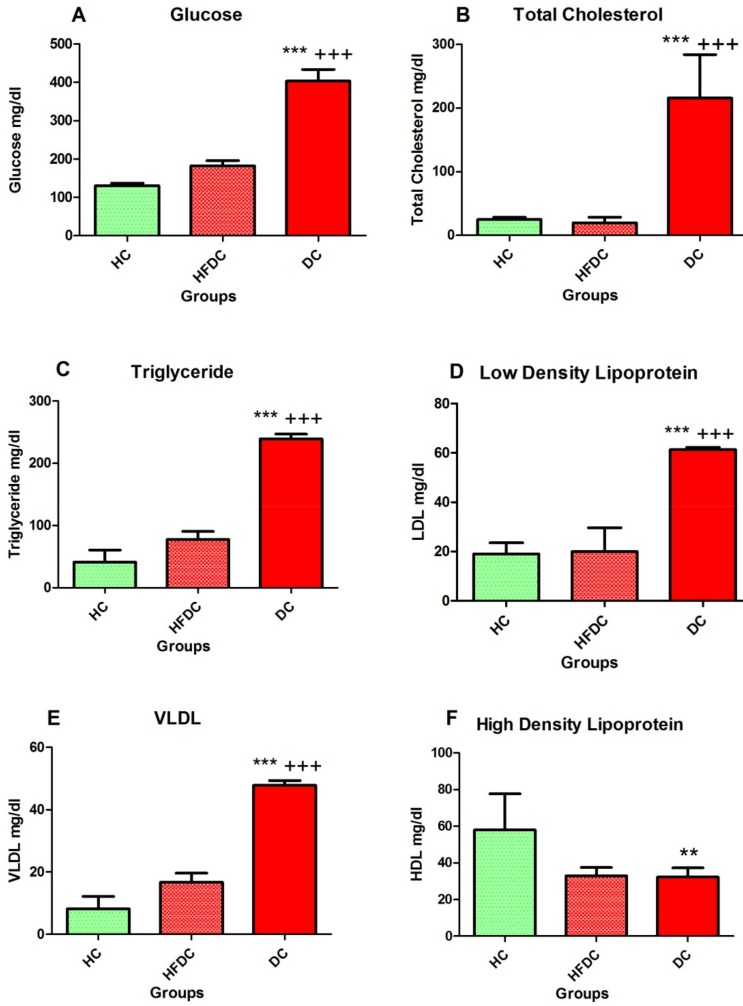
Diabetic rats showed elevated serum glucose and lipid profile. (**A**) Serum glucose levels; (**B**) Serum total cholesterol levels; (**C**) Serum triglyceride levels; (**D**) Serum low density lipoprotein levels; (**E**) Serum VLDL levels; (**F**) Serum high density lipoprotein levels. Results are recorded as a mean of three replicates and denoted as mean ± SD (*n* = 6 animals per group). ** *p* ≤ 0.01 and *** *p* ≤ 0.001, healthy control group; +++ *p* ≤ 0.001, HFD control group; (Dunnett’s Multiple Comparisons Test). HC: Healthy control, HFDC: High fat diet control, DC: Diabetes control. VLDL: Very low-density lipoprotein.

Serum glucose levels are shown in Figure 2A. In DC group, significant increases (*p* ≤ 0.001) in the serum glucose levels were observed as compared to the healthy (HC) and high fat diet (HFDC) control groups.

#### 3.5.2. Diabetic Rats Showed Elevated Serum Lipid Profile

Figure 2B–F depict serum lipid profiles of HC, HFDC and DC groups. Serum TC, TGs, LDL and VLDL levels were significantly (*p* ≤ 0.001) increased in diabetic group as compared to HC and HFDC groups. High density lipoprotein (HDL) was significantly higher (*p* ≤ 0.01) in HC group compared to the HFDC and DC groups.

#### 3.5.3. Elevation of Hepatic Enzymes in the Diabetic Rat Model

**Figure 3:**
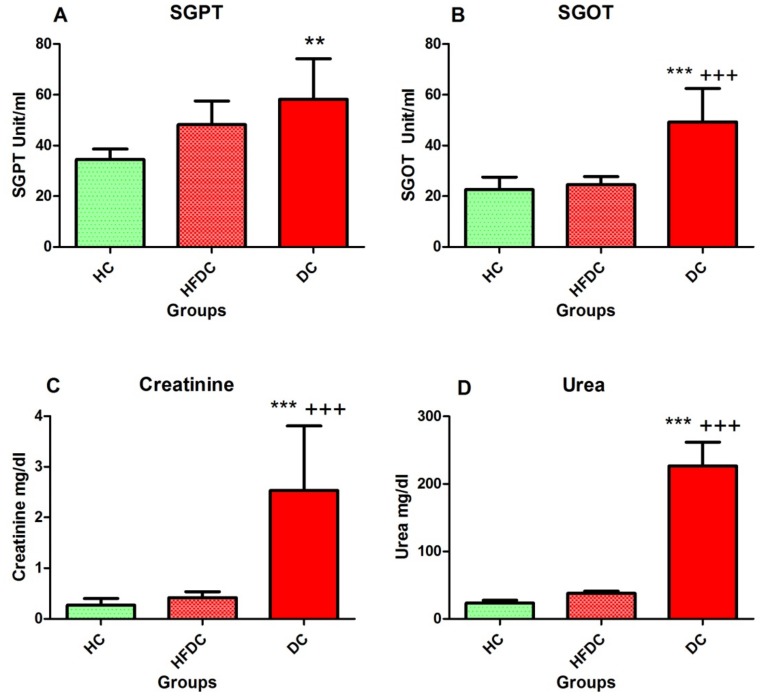
Assessment of liver and kidney function. (**A**) Serum SGPT levels; (**B**) Serum SGOT levels; (**C**) Serum creatinine levels; (**D**) Serum urea levels. Results are recorded as the mean of three replicates and denoted as mean ± SD (*n* = 6 animals per groups). ** *p* ≤ 0.01 and *** *p* ≤ 0.001, healthy control group; +++ *p* ≤ 0.001, HFD control group; (Dunnett’s Multiple Comparisons Test). HC: Healthy control, HFDC: High fat diet control, DC: Diabetes control. SGPT: Serum glutamic pyruvic transaminase; SGOT: Serum glutamic oxaloacetic transaminase.

Figure 3A,B shows liver function tests of HC, HFDC and DC groups. DC group showed significantly (*p* ≤ 0.01) increased serum glutamic pyruvic transaminase (SGPT) levels as compared to the HC and HFDC groups. In addition, serum glutamic oxaloacetic transaminase (SGOT) levels increased in DC as compared to HC (*p* ≤ 0.001) and HFDC groups.

#### 3.5.4. Assessment of Kidney Function

Serum creatinine and urea levels are depicted in Figure 3C–D. In DC group, serum creatinine and urea levels increased significantly (*p* ≤ 0.001) as compared to the HC and HFDC group.

### 3.6. Expression Profiles of Fatty Acid Metabolism Genes, Inflammatory Marker, and Related Transcription Factors from Liver

Expression of three transcription factors regulating six fatty acid metabolism genes and one inflammatory marker was studied in diabetic rats. Efficiencies of qRT-PCR amplifications of selected genes are depicted in Table S4.

#### 3.6.1. Alteration in Transcription Factors Expression Results in Lipid Abnormalities

**Figure 4:**
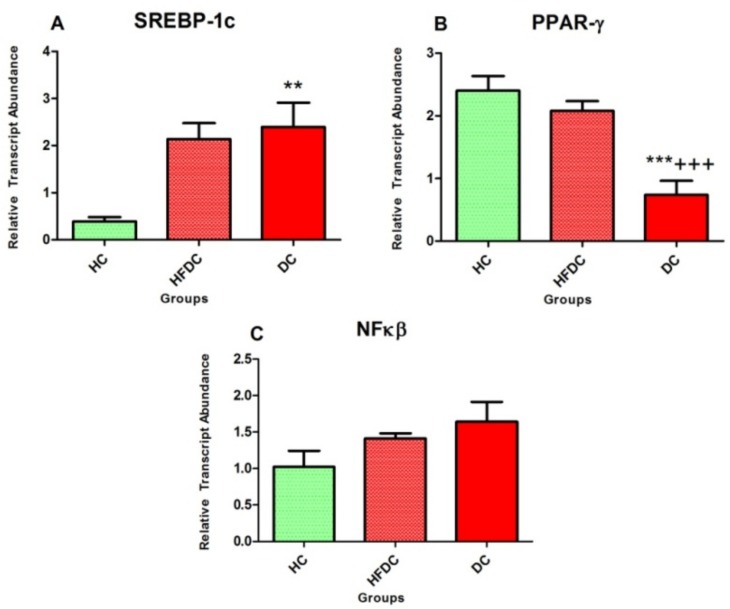
Alteration in transcription factors expression results in lipid abnormalities. Animals from the HC group were fed normal diet while HFDC and DC groups were on high fat diet. DC group was treated with STZ 35 mg/kg b.w. (ip). (**A**–**C**) represent the expression profiles of transcription factors *SREBP-1c*, *PPAR-γ* and *NFκβ* genes, respectively. Results are denoted as mean ± SD (*n* = 3 and the reactions were performed in duplicates). ** *p* ≤ 0.01 and *** *p* ≤ 0.001 when compared with the HC group and +++ *p* ≤ 0.001 when compared with the HFDC group (Dunnett’s multiple comparison test). HC: Healthy control, HFDC: High fat diet control, DC: Diabetic control group-treated STZ, *SREBP-1c*: Sterol Regulatory Element Binding Proteins-1c, *PPAR-γ*: Peroxisome Proliferator-Activated Receptor Gamma, *NFκβ*: Nuclear Factor-κβ.

Figure 4 represents the expression profile of transcription factors involved in regulation of fatty acid metabolism genes. In diabetic rats, hepatic expression of *SREBP-1c* was increased by ~4.6 and ~1.1 fold as compared to the healthy (*p* ≤ 0.01) and high fat diet control rats, respectively. At the same time, *PPAR-γ* expression was significantly (*p* ≤ 0.001) downregulated in DC group by ~0.3 and ~0.4 fold, as compared to the HC and HFDC groups. The hepatic expression of *NFκβ* was non-significantly up regulated by ~1.6 and ~1.2 fold in the DC group as compared to the HC and HFDC groups, respectively.

#### 3.6.2. Modulation of Expression in Fatty Acid Metabolism Genes

**Figure 5:**
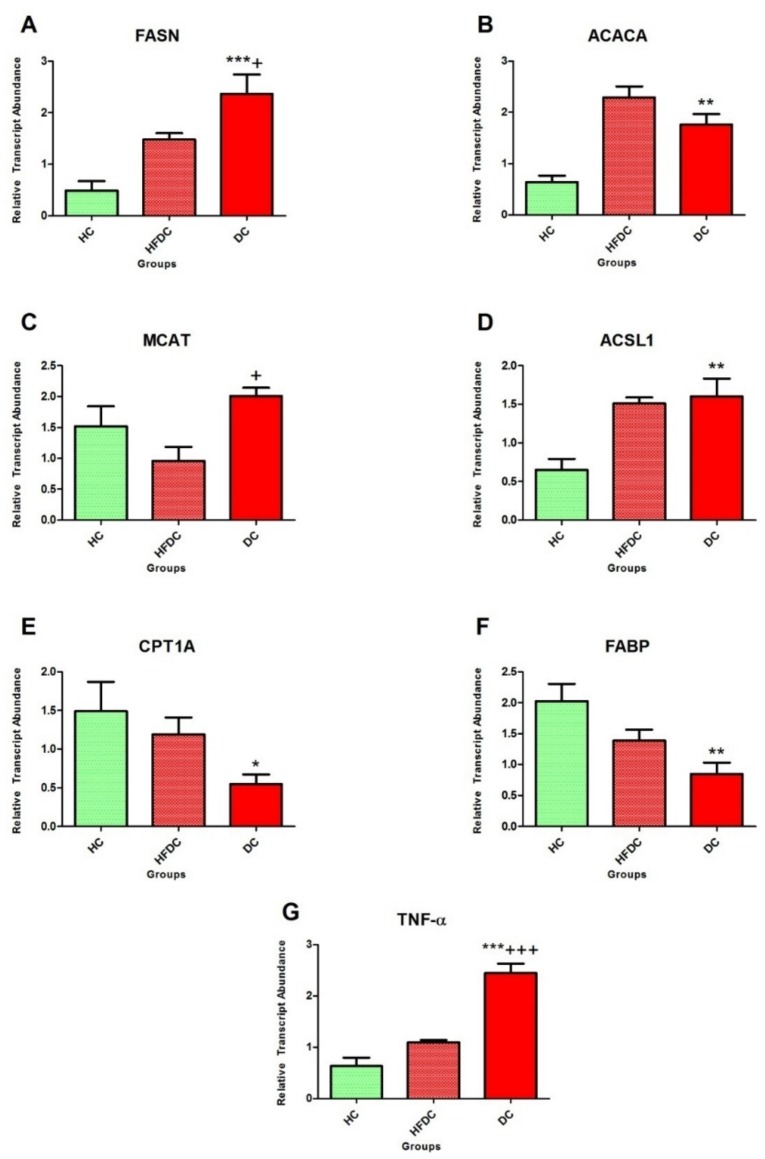
Modulation of expression in fatty acid metabolism and inflammatory genes. Animals from the HC group were fed on normal diet while HFDC and DC groups were on high fat diet. DC group was treated with STZ 35 mg/kg body weight (ip). (**A**–**F**) shows expression profiles of the *FASN*, *ACACA*, *MCAT*, *ACSL1*, *CPT1A*, *FABP* genes of fatty acid metabolism and Figure G shows expression of the *TNF-α* gene. Results are denoted as mean ± SD (*n* = 3 and the reactions were performed in duplicates). * *p* ≤ 0.05, ** *p* ≤ 0.01 and *** *p* ≤ 0.001 when compared with the HC group and + *p* ≤ 0.05, +++ *p* ≤ 0.001 when compared with the HFDC group (Dunnett’s multiple comparison test) HC: Healthy control, HFDC: High fat diet control, DC: Diabetic control group-treated STZ, *FASN*: Fatty Acid Synthase, *ACACA*: Acetyl-CoA Carboxylase Alpha, *MCAT*: Malonyl CoA: ACP Acyltransferase, *ACSL1*: Acyl-CoA Synthetase Long-chain family member 1, *CPT1A*: CarnitinePalmitoyltransferase 1A, *FABP*: Fatty-acid-binding proteins, *TNF-α*: Tumor necrosis factor-alpha.

Expression profile of fatty acid metabolism genes is depicted in Figure 5A–F. In diabetic rats, the expression levels of *FASN* were significantly up regulated by ~4.9 and ~1.6 fold as compared to HC (*p* ≤ 0.001) and HFDC (*p* ≤ 0.05) rats, respectively. In DC animals, *ACACA* gene expression increased by ~2.8 fold as compared to HC (*p* ≤ 0.01), while it decreased by ~0.7 fold as compared to the HFDC animals.

*MCAT* expression was upregulated by ~1.3 and ~2.1 fold as compared to the HC and HFDC (*p* ≤ 0.05) rats, while, *ACSL1* gene expression was upregulated in diabetic rats by ~1.2 and ~1.1 fold as compared to the HC (*p* ≤ 0.01) and HFDC rats, respectively.

Expression levels of *CPT1* and *FABP* genes showed similar trend. In diabetic rats, both genes were significantly downregulated by ~0.4 fold, as compared to the HC rats. Meanwhile, non-significant downregulation of *CPT1A* and *FABP* expression (by ~0.5 and ~0.6 fold, respectively) was observed as compared to the HFDC group.

#### 3.6.3. Expression of the Inflammatory Marker in the Experimental Groups

Figure 5G represents the expression profile of inflammatory marker, *TNF-α*. In diabetic rats, expression of *TNF-α* was significantly (*p* ≤ 0.001) upregulated by ~3.9 and ~2.2 fold as compared with the HC and HFDC rats, respectively.

### 3.7. Histological Examination of Liver, Kidney and Pancreas

**Figure 6:**
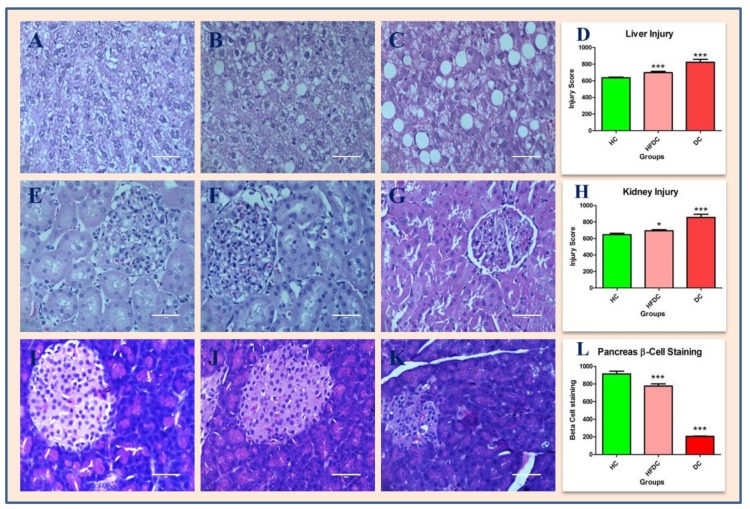
Histopathological examination of liver, kidney and pancrease tissues (40×, Scale bar = 50 µm). Histology of liver of healthy control (**A**), High fat diet (**B**), Diabetic control (**C**) and Liver injury (**D**); kidney of healthy control (**E**), High fat diet (**F**), Diabetic control (**G**) and kidney injury (**H**) and pancrease of healthy control (**I**), High fat diet (**J**), Diabetic control (**K**) and Beta cell staining. Tissue injury and beta cell staining data graphically expressed as mean ± SD and compared using Dunnett’s multiple comparison test against a healthy control (* *p*≤ 0.05 and *** *p* ≤ 0.001) (**D**,**H**,**L**).

Animals from DC group developed characteristic changes in liver, kidney and pancreas. Figure 6A–C shows H and E stained cross sections of paraffin-embedded livers of HC, HFDC and DC groups. HC group rats show normal architecture of hepatocytes. HFDC rats showed focal fatty changes, while DC rats showed microvesicular fatty changes in liver.

Figure 6E–G shows H and E stained cross sections of paraffin-embedded kidney for HC, HFDC and DC groups. HC and HFDC group rats showed normal architecture of kidney, while DC group rats showed vacuolated cytoplasm in distal convoluted tubules and in the medulla region.

Figure 6I–K show H and E stained cross sections of paraffin-embedded pancreatic tissues of HC, HFDC and DC group rats. HC and HFDC group showed normal architecture of pancreatic tissues. In DC group rats, size and number of islets of Langerhans and β cells were reduced. In diabetic rats, liver and kidney injury was siganificantly higher (*p* ≤ 0.001) as compared to healthy rats (Figure 6D,H). The number of β cells were decreased in diabetic group as compared to healthy group (Figure 6L).

## 4. Discussion

The hallmarks of type 2 diabetes are hyperglycemia, insulin resistance and insulin deficiency. Insulin resistance contributes to the characteristic dyslipidemia associated with T2DM [10] which is linked with significant cardiovascular incidences [10]. It accounts for nearly half of the diabetic dyslipidemic population [26]. Despite significant advances towards the prevention and management of diabetes in recent years, this chronic illness still has an alarmingly high rate of morbidity and mortality [12]. Hence, as an urgent health concern, aggressive management of abnormal lipid profile is highly recommended for patients affected by T2DM [10,27,28,29,30]. Initial preclinical assessment using animal models may assist in the implementation of preventive measures as well as development of management strategies for diabetes by mimicking its pathogenesis in humans.

Zucker diabetic fatty (ZDF) rat and db/db mouse are genetic models which develop diabetes spontaneously, resembling human pathology closely. However, development of diabetes in these models unlike in humans is mainly genetically determined [17,31]. In addition, these animal models are expensive and not readily available for research and regular screening experimentations. Furthermore, in the induced diabetic models most animals require relatively high dose of streptozotocin (STZ; >50 mg/kg) [17,19,32], which triggers characteristic features of type 1 diabetes more than type 2 diabetes. High fat diet and STZ models are routinely employed for studying diabetes and its associated complications in rats [8,15,20]. In the present study, HFD diet developed insulin resistance and glucose intolerance in the animals. Further, low dose STZ treatement causes distruction of few pancreatic beta cells. Together this leads to development of T2DM associated dyslipidemia. Thus, the current model is a hybrid between both diabetes etiologies (metabolic syndrome and STZ). Srinivasan et al. [8] used lard oil and other ingredients in HFD preparation. Binh et al. [20] also used complex HFD diet containing lard oil for 24 weeks. Plant-originated fats along with cholesterol have also been used to develop diabetic model for 10 weeks duration [15]. In our study, marked lipid abnormalities were observed compared to these previous reports. Recent studies further indicate that elevated serum cholesterol, LDL, triglyceride-rich lipoproteins and their remnants with simultaneous reduction in HDL constitute major risk factors for cardiovascular disease (CVD) in individuals with T2DM [9,33,34]. In the present animal model, significantly disturbed lipid profile followed by development of glucose intolerance was observed.

We assessed glucose tolerance by IPGTT after eight weeks. All HFD fed animals showed higher baseline fasting glucose and lower glucose clearance rate than HC animals (Figure 1). This might be the outcome of insulin resistance. HFD fed rats also showed increased body weight as compared to the HC group (Figure S2). This might be due to the intake of high energy diet, containing saturated fats (lard oil) and deposition of these fats as abdominal adipose tissue. Our results are in agreement with previous reports [35,36].

The detrimental effect of continuously elevated serum glucose and lipid levels on various organs including liver, kidney and pancreas is well documented [20,37]. Liver and kidney are the most commonly affected organs [37] exhibiting glycogen deposition, steatosis, fibrosis among other characteristics. Hence, a functional test for these two organs has to be considered in the development of an ideal animal model for diabetes. In the present study, serum SGOT, SGPT, creatinine and urea levels were elevated. Similarly, in histopathology analysis, fatty liver changes and vacuolated cytoplasm in kidney tubules were detected.

To date, various rat models have been reported for the study of diabetes and its complications [8,15,17,19,20,31]. Similar studies have been also conducted in guinea pigs [3]. However, none of these animal models are validated at molecular levels. To the best of our knowledge, only a single study has been published to date reporting on the molecular changes in the animal model of diabetes [38]. This study reports the expression of adipokines and their receptors. An ample amount of reports are available for genes and their regulatory elements involved in fatty acid metabolism and inflammation have been implicated in diabetes [39,40]. We found that, in our animal model, these genes together with their regulatory elements that were involved in fatty acid metabolism and inflammation are associated with diabetes. Thus, the present study reports a potential mechanism of diabetes development in the HFD animal model.

*SREBP* is a transcription factor which plays an important role in the regulation of fatty acid and cholesterol metabolism [39]. *SREBP-1c* is expressed relatively abundant in liver and its overexpression leads to increased production of TGs [41] Hepatic steatosis (fatty liver) is associated with increased expression of *SREBP-1c* [42]. In the present study, *SERBP-1c* expression, which results in increased serum cholesterol and triglycerides was found to be upregulated in HFD fed rats compared to healthy animals. Distinct histologic features of fatty liver were also evident.

*SREBP* is involved in the regulation of various lipogenic genes such as *ACACA*, *FASN*, *ACSL1* and *MCAT* which are found to be overexpressed in diabetes [43]. In our experiments, expression of *SREBP-1c* and consequently, *FASN*, *ACACA*, *ACSL1* and *MCAT* expression was also found to be upregulated. *CPT1A* and *FABP* genes are involved in the transportation of fatty acids and beta oxidation. In the present study, these genes were downregulated as compared to the healthy control. This reduced expression may be one reason for the increased lipid abnormalities observed in the diabetic rats models.

Similarly, *PPAR-γ* is involved in the fatty acid and energy metabolism [44], as well as in glucose homeostasis, whereby the insulin-stimulated glucose uptake by peripheral tissues are increased while hepatic gluconeogenesis is reduced [44]. Activation of *PPAR-γ*, stimulates β-oxidation of fatty acids, thereby decreasing serum TG levels and increasing HDL cholesterol [45,46]. Our results further support previous findings [44,45,46].

*NF-κβ* regulates inflammatory markers and is involved in the pathogenesis of insulin resistance as well as type 2 diabetes mellitus [40,47]. High levels of pro-inflammatory cytokines such as *TNF-α* are also linked with insulin resistance and T2DM [48]. In spite of its role in inflammation, *TNF-α* increases lipogenesis and lipolysis, thus contributing to lipid abnormalities such as hypertriglyceridemia [49,50]. As also shown by our results, these findings indicate that upregulation of *TNF-α* might play a role in progression of dyslipidemia in T2DM.

*FABP* is a cytoplasmic lipid transporter which is involved in insulin resistance, progression of dyslipidemia and atherosclerosis [51]. Newberry et al. [52] reported that in *L-FABP*-null mice, serum TG level was increased due to reduced accumulation of triglyceride in liver. In normal condition, *FABP* facilitates fatty acid oxidation in the liver [53]. Downregulation of *L-FABP* in diabetic rats may potentially account for increased TG levels.

## 5. Conclusions

Thus, the proposed model in this study results in the development of profound diabetic dyslipidemia and is found to be also suitable for the practical investigations and testing of different compounds for the effective management of T2DM and its complications. The model is validated at biochemical, molecular and histopathological levels for diabetes and its associated complications. The observed pathophysiology mimics the metabolic characteristics of human T2DM. This novel animal model can be used for screening new therapeutics for the effective management of diabetes mellitus.

## Figures and Tables

**Table 1:** Estimation of serum glucose and insulin.

Experimental Groups	Glucose (mM)	Insulin (µU/mL)
Healthy Control (HC)	3.91 ± 0.08	8.30 ± 0.07
High Fat diet Control (HFDC)	5.65 ± 0.17 ^***^	9.52 ± 0.16 ^**^
Diabetic Control (DC)	5.51 ± 0.04 ^***^	10.12 ± 0.30 ^***^

Results are recorded as the mean of three replicates and denoted as mean ± SD (*n* = 6 animals per groups). ** *p* ≤ 0.01; *** *p* ≤ 0.001, HFDC and DC group are compared with the healthy control group (Dunnett’s Multiple Comparisons Test).

## Supplementary Materials

The following are available online at https://www.mdpi.com/2227-9059/7/4/76/s1, Table S1. High Fat Diet (HFD) composition; Table S2. Primer sequences of selected RT-PCR genes; Table S3. Biochemical estimation before initiation of HFD diet; Table S4. The efficiency of qRT-PCR for relative quantification of mRNA. Efficiency is calculated from the slope of the curve as E = 10(−1/slope)^−1^; Figure S1. HFD with 30% lard oil fed rats showed glucose intolerance; Figure S2. Animals weights of experimental groups during experiment
