# Supplementary material for: High Energy Intake Induced Overexpression of Transcription Factors and Its Regulatory Genes Involved in Acceleration of Hepatic Lipogenesis: A Rat Model for Type 2 Diabetes
Source: Biomedicines. 2019 Sep 27;7(4):76. doi: 10.3390/biomedicines7040076 (PMC6966540; doi:10.3390/biomedicines7040076)
Supplement: Supplementary file 1 [file biomedicines-07-00076-s001.pdf]

Supplementary Table

Table S1. High Fat Diet (HFD) composition

Ingredients	Weight in gm (per Kg)
Powdered normal pellet diet [Nutrivet Life Science, Pune, M.S., India]	700
Lard oil [Pune, M.S., India]	300

Table S2. Primer sequences of selected RT-PCR genes

S.No.	Target Genes	Primers	Sequence
		**Housekeeping Gene**	
1	*Glyceraldehyde-3-phosphate dehydrogenase*	Forward	AGTTCAACGGCACAGTCAAG
		Reverse	TACTCAGCACCAGCATCACC
		**Transcription Factors**	
1	*Sterol Regulatory Element-Binding Proteins*	Forward	AAACCTGAAGTGGTAGAAAC
		Reverse	TTATCCTCAAAGGCTGGG
2	*Peroxisome Proliferator-Activated Receptor Gamma*	Forward	AAGACAACAGACAAATCACC
		Reverse	CAGGGATATTTTTGGCATACTC
3	*Nuclear Factor-κβ*	Forward	AAAAACGAGCCTAGAGATTG
		Reverse	ACATCCTCTTCCTTGTCTTC
		**Fatty Acid Metabolism Genes**	
1	*Fatty Acid Synthase*	Forward	AAAAGGAAAGTAGAGTGTGC
		Reverse	GACACATTCTGTTCACTACAG
2	*Acetyl-CoA Carboxylase Alpha*	Forward	AGCAGTATTTGAACACATGG
		Reverse	CAGTTCCAAGAAGTAGAAGC
3	*Malonyl CoA: ACP Acyltransferase*	Forward	AAAACTCTAGGCTCAATCAAC
		Reverse	GGATGTGTGTATTTATGCCC
4	*acyl-CoA Synthetase Long-chain family member 1*	Forward	ACATTATGAACGATTGCTCC
		Reverse	GCATTACACACTCTACAACG
5	*Carnitine Palmitoyltransferase 1A*	Forward	CACTGATGAAGGAAGAAGAC
		Reverse	CCAGTCACTCACGTAATTTG
6	*Fatty Acid Binding Protein*	Forward	TGGAGGGTGACAATAAAATG
		Reverse	TCATGGTATTGGTGATTGTG

		**Inflammatory Marker**	
1	*Tumor Necrosis Factors-α*	Forward	CTCACACTCAGATCATCTTC
		Reverse	GAGAACCTGGGAGTAGATAAG

Table S3. Biochemical estimation before initiation of HFD diet

Biochemical parameters	Healthy control (HC)	High fat diet control (HFDC)	Diabetic control (DC)
Glucose (mg/dl)	96.82 ± 11.24	95.75 ± 17.18	89.13 ± 7.23
**Lipid profile (mg/dl)**			
Total cholesterol	53.00 ± 15.31	70.57 ± 18.04	47.28 ± 13.63
Triglycerides	7.350 ± 2.34	11.00 ± 6.30	7.367 ± 1.12
HDL- cholesterol	12.23 ± 6.95	21.20 ± 7.70	22.18 ± 12.92
LDL- cholesterol	26.75 ± 5.74	19.58 ± 6.07	25.67 ± 10.80
VLDL- cholesterol	1.450 ± 0.48	1.600 ± 0.72	1.483 ± 0.22
**Liver Function Test (Unit/ml)**			
SGOT	39.97 ± 6.66	24.58 ± 3.02	32.88 ± 24.07
SGPT	30.13 ± 6.56	32.45 ± 3.82	29.85 ± 13.28
**Kidney Function Test (mg/dl)**			
Creatinine	1.167 ± 0.68	0.7167 ± 0.19	0.9500 ± 0.51
Urea	29.87 ± 10.19	27.78 ± 3.27	27.62 ± 4.34

Results are recorded as the mean of three replicates and denoted as mean ± SD (n=6 animals per groups). All values for HFDC and DC groups were non-significantly different as compared with the healthy control group (Dunnett Multiple Comparisons Test). LDL: Low-density lipoprotein, VLDL: Very low-density lipoprotein, HDL: High-density lipoprotein, SGOT: Serum glutamic oxaloacetic transaminase, SGPT: Serum glutamic pyruvic transaminase.

Table S4. The efficiency of qRT-PCR for relative quantification of mRNA. Efficiency is calculated from the slope of the curve as E = 10(−1/slope)$^{-1}$

Sr.No.	Target Genes	Symbol	Efficiency
	**Housekeeping Gene**		
1	*Glyceraldehyde-3-phosphate dehydrogenase*	*GAPDH*	86.29
	**Transcription Factors**		
1	*Sterol Regulatory Element-Binding Proteins-1c*	*SREBP-1c*	95.60
2	*Peroxisome Proliferator-Activated Receptor Gamma*	*PPAR-γ*	104.66
3	*Nuclear Factor-κβ*	*NF-κβ*	83.88
	**Fatty Acid Metabolism Genes**		
1	*Fatty Acid Synthase*	*FASN*	80.56
2	*Acetyl-CoA Carboxylase Alpha*	*ACACA*	86.79
3	*Malonyl CoA: ACP Acyltransferase*	*MCAT*	85.54
4	*acyl-CoA Synthetase Long-chain family member 1*	*ACSL 1*	86.26
5	*Carnitine Palmitoyltransferase 1A*	*CPT 1A*	88.57
6	*Fatty Acid Binding Protein*	*FABP*	87.59
	**Inflammatory Markers**		
1	*Tumor Necrosis Factors-α*	*TNF-α*	100.79

S

upplementary Figure

Figure S1. HFD with 30 % lard oil fed rats showed glucose intolerance

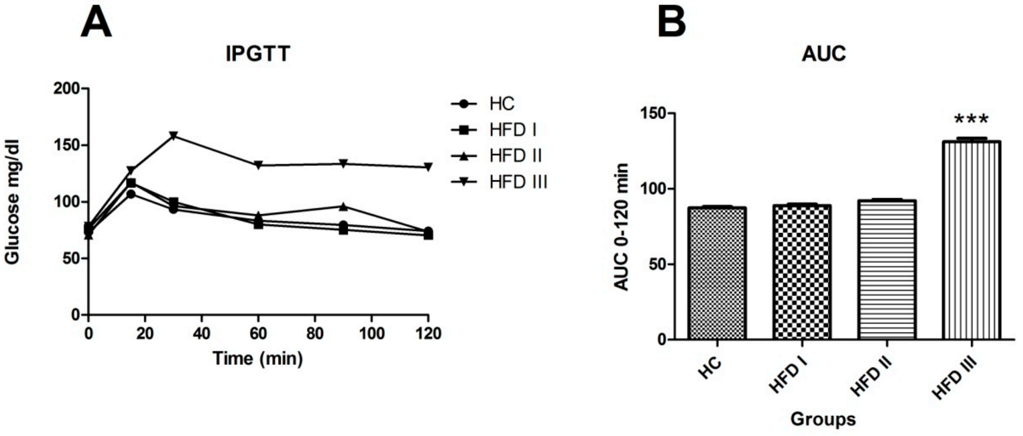

A: Variations in glucose levels during IPGTT

B: Area under curve (AUC) for IPGTT. Results are recorded as the mean of three replicates and denoted as mean ± SE (n=6 animals per groups). ***p≤0.001, when compared with the healthy control group (Dunnett Multiple Comparisons Test). HC: Healthy control, HFD I: 10 % lard oil, HFD II: 20 % lard oil and HFD III: 30% lard oil group.

Figures S2. Animals weights of experimental groups during experiment

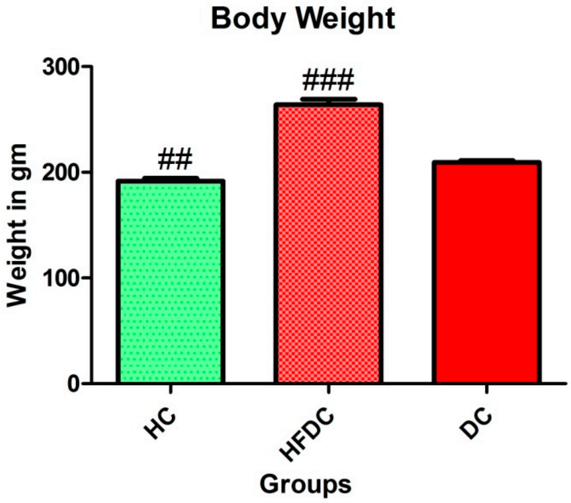

Results are recorded as the mean of three replicates and denoted as mean ± SE. ##p≤0.01 and ###p≤0.001, when compared with the diabetic control group (Dunnett Multiple Comparisons Test). HC: Healthy control, HFD: High fat diet control, DC: Diabetes control.
